# Culturable Airborne Bacteria in Outdoor Poultry-Slaughtering Facility

**DOI:** 10.1264/jsme2.ME12178

**Published:** 2013-03-09

**Authors:** Ruiping Liang, Peng Xiao, Ruiping She, Shiguo Han, Lingling Chang, Lingxiao Zheng

**Affiliations:** 1 Department of Veterinary Public Health, Key Laboratory of Zoologies of Ministry of Agriculture, College of Veterinary Medicine, China Agricultural University, Beijing, 100193, China; 2 Beijing Huadu Broiler Corporations, Beijing 102211, China

**Keywords:** culturable airborne bacteria, bioaerosol, BIOLOG, dominant genus

## Abstract

Airborne bacteria are important biological components of the aerosols and have a close relationship with human health as they can have adverse effects through infection and toxicity; higher concentrations can result in various microbial diseases. Moreover, they have a great influence on air quality in Beijing. In this study, a systematic survey on culturable airborne bacteria was carried out for 1 year at a slaughtering plant in Beijing. Bacterial samples were collected with FA-1 sampler for 3 min, three times each day, for three consecutive days of each month from three sampling sites using BIOLOG identification technology. Results showed that Gram-positive bacteria contributed 80%–85% and were much more prevalent than Gram-negative bacteria. Amongst 47 genera of bacteria, including 31 Gram-positive bacteria and 16 Gram-negative bacteria, *Micrococcus*, *Staphylococcus*, *Bacillus*, *Corynebacterium*, and *Pseudomonas* were dominant, and *Micrococcus*, which contributed 20%–30%, was the most dominant genus. The concentration of airborne bacteria was significantly higher in shed used to stay chicken waiting for slaughtering (SSC) and entrances to personnel and transport vehicles with products (EPV) than in green belt (GB). During the year, bacterial concentrations in summer and autumn were much higher than in winter and spring in SSC and EPV, and there were no significant variations in bacterial concentrations in GB. In different periods, a lower concentration of airborne bacteria was found at 13:00.

Airborne bacteria are among the most common organisms in nature. They are considered to be correlated with air pollution and have been proposed as a cause of adverse health effects on humans (10). Elevated levels of particle air pollution have been associated with upper respiratory irritation, chronic bronchitis, organic dust toxin syndrome, or other respiratory symptoms (2).

Modern agriculture activities have changed our living environment. High microorganism pollution is a feature of many commercial slaughtering facilities. The numbers and types of microorganisms that are either bound to slaughtering facility dust or are individually airborne may play a contributing role; however, no previous studies have examined the microbial composition of a poultry slaughtering facility. Regarding their adverse effects on human health, many studies have been carried out. It was reported that airborne bacteria in pig confinement buildings in the United States, Canada, The Netherlands, Sweden, and Poland reach levels of 10^5^ to 10^6^ CFU m^−3^ (1, 3, 4, 6, 8, 11) and up to 10^7^ CFU m^−3^ in the United Kingdom (5). Gram-positive bacteria are the predominant bacteria present (1, 3, 5–7, 11, 16) and microbial genera have been identified in many swine confinement units (3–5, 7, 8). Some organisms are recognized as potential agents inducing extrinsic allergic alveolitis and causing pathogenic infection (4). The study showed that the highest median concentration of total (2.0×10^7^ cells m^−3^) bacteria was found in poultry houses in Switzerland (13). In subtropical Taiwan, the study quantified the levels of airborne microorganisms in six swine farms with more than 10,000 pigs, and the results showed that mean concentrations of culturable bacteria and Gram-negative bacteria were 3.3×10^5^ CFU m^−3^ and 143.7 CFU m^−3^, respectively (2). A total of 1,408 cattle held in eight commercial feedlot pens were used to examine the quantity and diversity of micro-organisms in cattle feedlot air (23). The results showed that only non-pathogenic Gram-positive organisms were recovered; however, Gram-negative bacteria may have been present in a viable but non-culturable state (23).

It is reported that the dominant bacteria were *Micrococcus*, *Staphylococcus*, *Bacillus* and *Pseudomonas* in the atmosphere (15, 18–20, 22), and their concentrations differed according to location because of local environmental variables, bacterial substrates, and human activities (9). It is important to survey both the level and distribution of airborne microorganisms systematically and extensively across different outdoor environments in a poultry slaughtering facility. Three sampling sites in different functional areas were selected for research on the community structure and dynamic change of culturable bacteria in the outdoor environment of a poultry slaughtering facility. The objectives of this study were (i) to describe the groups and concentrations of airborne culturable bacteria in the outdoor environment of a poultry slaughtering facility, and (ii) to reveal the distribution characteristics and dynamic changes of bacteria concentrations at three sampling sites in the outdoor environment of a poultry slaughtering facility.

## Materials and Methods

### Sampling sites

**Fig. 1:**
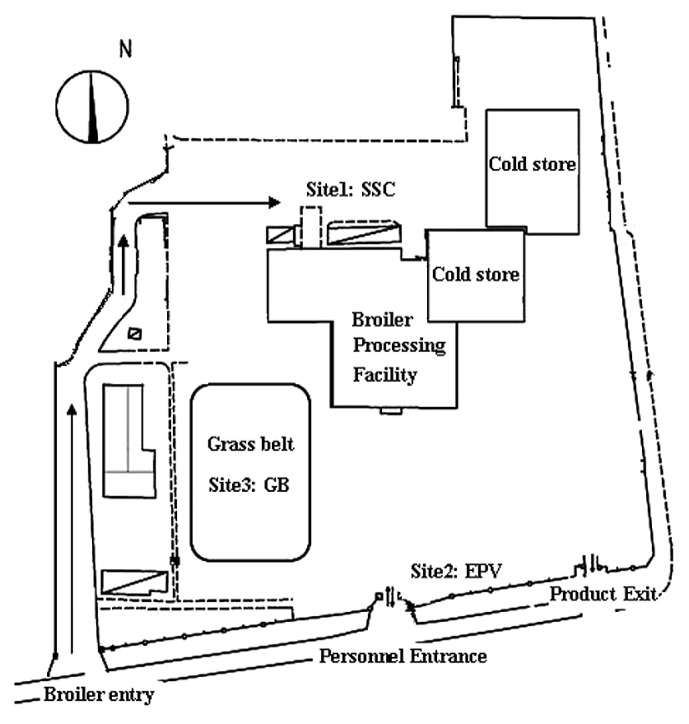
Map of the plant containing the sampling sites.

Air samples were collected from an outdoor poultry-slaughtering facility (73,000 birds slaughtered per day) situated in a suburban district of Chanping in Beijing, China, about 50 km from the city center. Beijing has a territorially monsoon climate and is situated in a warm temperate zone, which has a dry season from November to April and a wet season from May to October. This facility employs approximately 900 workers, operates 12 h d^−1^, and products 150 tons production d^−1^. The production is mainly exported to Japan, South Korea, Hong Kong, Central Asia, Canada, and Singapore. Three sites were selected for the study in this plant (Table 1; Fig. 1): (i) Shed used to stay chicken waiting for slaughtering (SSC), with a green area contributing to about 50% of the total area, and a few transport vehicles for chickens, with serious air pollution; (ii) Entrances to personnel and transport vehicles with products (EPV), with a small green area amounting to no more than 5% vegetation coverage and about 10 times h^−1^ flow of transport vehicles, and some personnel flow at commuting time, with a little air pollution; (iii) Green belt (GB), with a green area contributing to more than 95% of the total area and little flow of vehicles and personnel, with little air pollution.

### Sampling methods

A six-stage culturable FA-1 sampler (imitation Andersen sampler) (Applied Technical Institute of Liaoyang, China) was used to isolate culturable bacteria from the atmosphere. Each stage includes a plate with 400 holes of uniform diameter through which air is drawn at 28.3 L min^−1^ to contact with Petri dishes containing agar media. AT each sampling site, the sampler was mounted 1.5 m above ground level on a platform. Sampling was conducted from June 2011 to May 2012. Samples were collected for 3 min, three times (9:00, 13:00 and 17:00 hours) each day, and continued for three consecutive days of each month. For each sampling, the FA-1 sampler was loaded with 9.0 cm Petri dishes containing Nutrient agar to culture the collected bacterial samples. Exposed culture dishes were incubated for 48 h at 37°C. Results were then expressed as colony-forming units per cubic meter of air (CFU m^−3^). CFU m^−3^ was calculated as:

(Number of colonies×1,000)/(Sampling time×Velocity of air flow)

### Identification of bacteria

Colonies were picked, further purified, Gram-stained, examined by microscopy, identified by biochemical reactions and grouped into Gram-positive cocci, Gram-positive spore bacillus, Gram-positive bacteria, Gram-negative non-enteric bacteria, and Gram-negative intestinal bacteria. After a series of operation steps including purifying, Gram staining, enrichment culture, preparation of bacteria suspension and inoculating the prepared bacterial suspension into the 96 wells of the plate, Gram-positive bacteria were inoculated onto the GP plate of the 96 wells, Gram-negative bacteria were inoculated onto the GN plate of the 96 wells. They were cultured under suitable conditions according to the growth characteristics of the different types of bacteria, and then were identified to their species using the Biolog Microbial Identification System.

Biolog MicroPlates were used to test the ability of a micro-organism assimilating or oxidizing compounds from a preselected panel of different carbon sources. The test yielded a characteristic pattern of reddish-orange well changes, which constituted a “metabolic fingerprint”. All necessary nutrients and biochemicals were pre-filled and dried in the 96 wells of the plate. Iodonitrotetrazolium violet was used as a redox dye to colorimetrically indicate the mitochondrial activity that was stimulated during the oxidation of certain carbon sources. The MicroPlates were incubated for 16–24 h. The pattern of reddish-orange wells was read with the Biolog MicroStation Reader to detect and quantify color responses. Biolog’s MicroLog computer software automatically cross-referenced the pattern with an extensive library of species and genus. If an appropriate match was found, a presumptive identification of the isolate was made.

Biolog software can list 10 results in accordance with the matching degree of the reaction results of the 96 microplates in the database. Once the identification results match well with the database, the status bar will turn green. If the identification results are unreliable, the status bar will turn yellow and display “NOID”; however, the system will still list the 10 most likely results. Every result illustrates three important parameters, namely Probability (PROB), Similarity (SIM) and Distance (DIS). DIS and SIM are the most important values in the system; the DIS value represents the distance of testing results to the data bars in the corresponding database; the SIM value indicates the similarity of testing results to the data bars in the corresponding database. The Biolog Microbial Identification System (MIS) stipulates that when the bacterial culture time is 4–6 h, its SIM values are ≥0.75; when the culture time is 16–24 h, SIM values are ≥0.50; therefore, the system can automatically show the species name based on identification results. The results show higher reliability when the SIM value is closer to 1.00. When the SIM value is less than 0.5, and the SIM value of the same genus name sum in the identification results is higher than 0.5, it will automatically shows the results in the form of the genus name.

### Statistical analysis

Descriptive statistical analysis was performed using SPSS for Windows 10.0 (SPSS Inc., Chicago, IL, USA) by one-way analysis of variance (ANOVA).

## Results

### Bacterial groups

Forty-seven genera of culturable bacteria were identified from all sampling sites, including 31 Gram-positive genera at 66.0% and 16 Gram-positive genera at 34.0% (Table 2).

The dominant groups in 47 genera were *Micrococcus*, *Staphylococcus*, *Bacillus*, *Corynebacterium* and *Pseudomonas* successively. These five dominant groups occupied about 50% of the total culturable bacteria, with *Micrococcus* at 20–30% and *Pseudomonas* at 2.5–5.0%.

Within the dominant groups, *Micrococcus* had the maximum bacterial concentration percentage and accounted for 32.5% at the EPV, 26.6% in the SSC, and 20.5% in the GB, respectively.

*Staphylococcus* was the second most common group isolated from samples, followed by *Bacillus*, *Corynebacterium* and *Pseudomonas*. Their concentration percentages varied from 2.82% to 16.28%.

The concentration percentages of *Micrococcus* (*P*<0.05) and *Bacillus* (*P*<0.05) were significantly higher at EPV than in the GB and SSC; however, the concentration percentages of *Staphylococcus* (*P*<0.01) and *Pseudomonas* (*P*<0.05) were significantly higher in the GB than in the SSC and at the EPV, and no significant differences in concentration percentages in other groups were found at any sampling sites.

### Bacterial concentration

#### Overall concentration

Considering all sampling sites, the concentration range of culturable bacteria was 48–27,920 CFU m^−3^ and the mean and median were 2,330 CFU m^−3^ and 1,420 CFU m^−3^, respectively (Table 3). Significantly higher bacterial concentrations were found in the SSC and at the EPV than in the GB (*P*<0.05). The mean concentration was 2,714 CFU m^−3^ (142–17,876 CFU m^−3^) in the SSC, 2,664 CFU m^−3^ (188–27,920 CFU m^−3^) at the EPV and 1,582 CFU m^−3^ (48–10,658 CFU m^−3^) in the GB (Table 3).

**Fig. 2:**
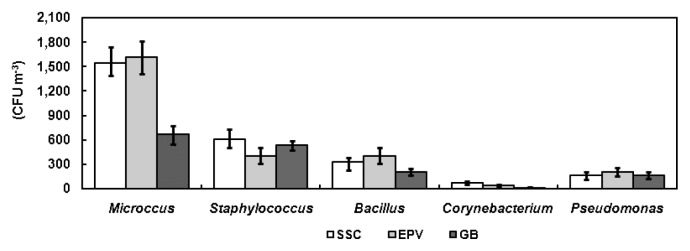
Dominant bacterial concentration at the three sampling sites.

The concentrations of *Micrococcus* and *Bacillus* in the SSC and at the EPV were higher than in the GB (*P*<0.01), but no significant difference was found between the SSC and the EPV (*P*>0.05). Concerning *Staphylococcus*, the lowest concentration was observed at the EPV (*P*<0.01); however, there were no significant differences in *Corynebacterium* and *Pseudomonas* concentrations at any sampling sites (*P*>0.05; Fig. 2).

#### Seasonal concentration

**Fig. 3:**
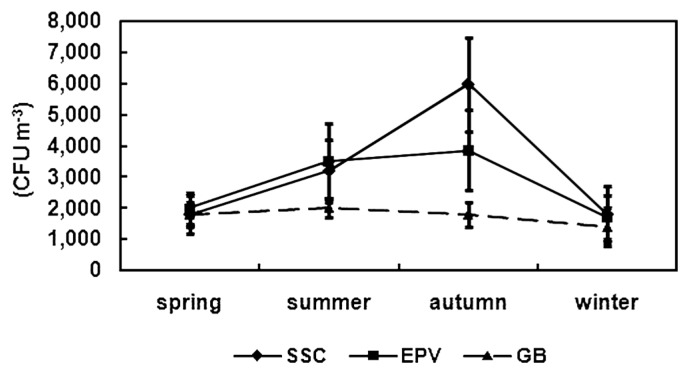
Seasonal variation patterns of the airborne concentration of total culturable bacteria.

Significant differences in bacterial concentrations among seasons existed in the SSC and the EPV, where the mean concentrations were higher in summer (June to August) and autumn (September to November), and lower in spring (March to May) and winter (December to February) (*P*<0.01), while no significant variation of bacterial concentrations was observed in different seasons in the GB (*P*>0.05). At the three sampling sites, there were no significant differences in bacterial concentrations between spring and winter (*P*>0.05). In the SSC and EPV, the concentration was highest during autumn (*P*<0.01), accounting for 5,975.3 CFU m^−3^ and 3,866.5 CFU m^−3^, respectively (Fig. 3).

#### Monthly concentration

**Fig. 4:**
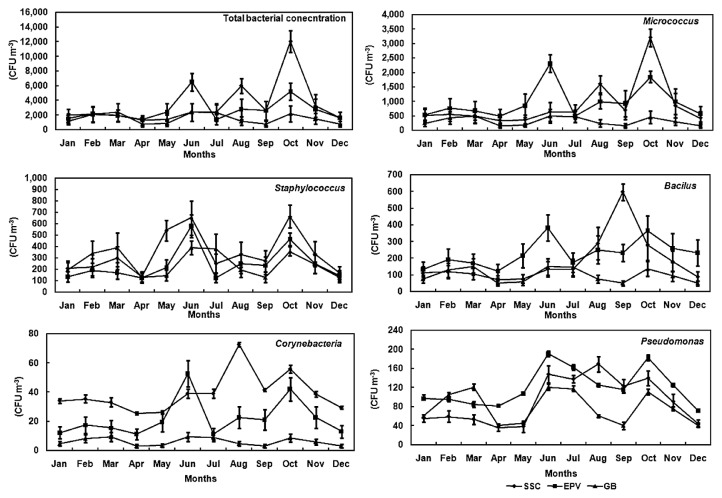
Monthly variation pattern of the airborne concentration of culturable bacteria at the three sampling sites.

The total bacteria concentrations from June to October were higher than in other months in the SSC and EPV (*P*<0.05; Fig. 4). The highest concentration was recorded in October (12,047 CFU m^−3^) in the SSC and in June (6,514 CFU m^−3^) at the EPV (Fig. 4). The lowest concentrations were found in April in the SSC (1,286 CFU m^−3^) and EPV (1,380 CFU m^−3^); however, the concentrations exhibited no significant differences at the EPV throughout the year. At all sampling sites, no significant variations in bacteria concentrations were found from November to May (*P*>0.05; Fig. 4). The same variations were observed between *Micrococcus* concentrations and total bacterial concentrations at the three sampling sites during the year. The highest concentration of *Micrococcus* was recorded in October (3,205 CFU m^−3^) in the SSC and in June (2,313 CFU m^−3^) at the EPV. The lowest concentration was found in April (342 CFU m^−3^) in the SSC and GB (490 CFU m^−3^). At all sampling sites, for *Staphylococcus*, the concentration levels in December and April were relatively lower than in other months; however, no significant variations in *Corynebacterium* and *Pseudomonas* concentrations were observed during the year at any sampling sites. The same variations were observed in *Bacillus* except for the higher concentration in September in the SSC (Fig. 4).

#### Diurnal changes at three sampling times

**Fig. 5:**
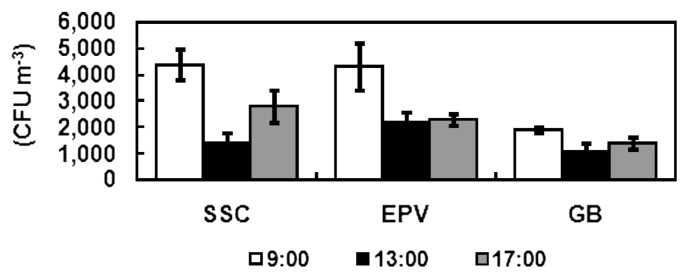
Diurnal changes of total bacterial concentration at three sampling times in a day.

**Fig. 6:**
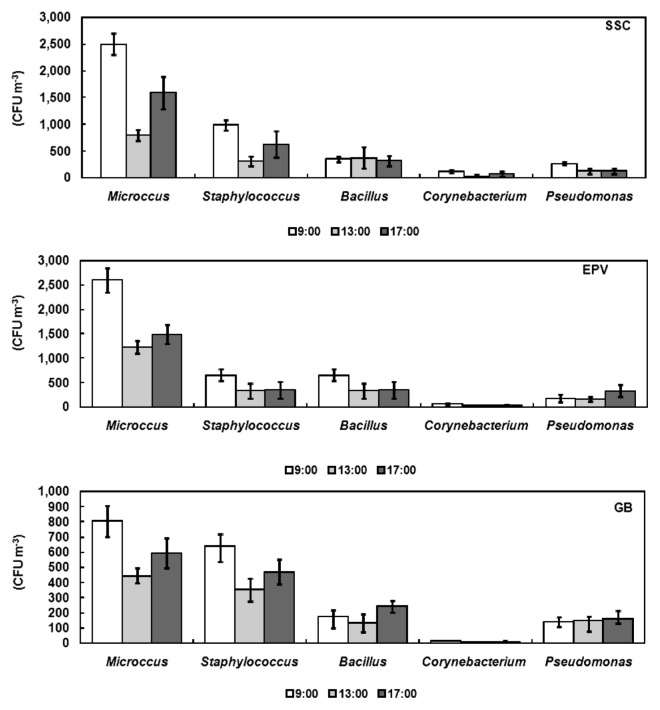
Diurnal changes of bacterial group concentration at three sampling times in a day.

Total bacterial concentration was lower at 13:00 than at 9:00 and 15:00 at different sampling sites (Fig. 5). In the SSC, the concentration was higher at 9:00 (*P*<0.05) and 17:00 (*P*<0.05) than at 13:00. At the EPV, the concentration was higher at 9:00 (*P*<0.05) than at 13:00. No significant difference at the three sampling times existed in the GB (*P*>0.05). The highest concentrations of *Micrococcus* were recorded at 9:00 (*P*<0.05), and the lowest concentration of *Staphylococcus* was found at 13:00 at all sampling sites (*P*<0.05; Fig. 6).

## Discussion

In general, the bacterial concentration in the slaughtering facility was higher than in studies conducted in other functional areas (21), which could lead to serious microbial pollution in the atmosphere. The bacterial concentrations in the atmosphere varied greatly at different sampling sites during a year at the plant, and the wide range of concentrations could be attributed to the micro-environmental and meteorological conditions, sampling time during the day and year, and different climatic conditions during the year (17). In other studies, there were large differences in total bacterial concentrations and marked variations were also found in cities in other countries (2, 14). For example, Katja *et al.* (13) reported the highest median concentrations of total (2.0×10^7^ CFU m^−3^) and bacteria (4.4×10^5^ CFU m^−3^) in Swiss poultry houses. These marked differences could be attributed to the geographic location, different bacterial growth substrates in different countries, as well as different types of sampler and media and different sampling methods used by the researchers (10).

The prevalent bacterial groups from all the sampling sites were *Micrococcus*, *Staphylococcus*, *Bacillus*, *Corynebacterium* and *Pseudomonas*, some of which have been reported as the most common airborne bacteria in different environments in other studies (10, 12, 13, 25). *Micrococcus*, comprising more than one third of the collected samples, was the most dominant bacterial group in the present study; however, it was reported that *Staphylococcus* and *Bacillus* were the most dominant bacterial group in open-air swine houses in Spain (14).

A significantly high concentration of airborne bacteria was observed in SSC and EPV in summer and in autumn at this plant. On one hand, air temperature and moisture in the microenvironment in summer and autumn could be adaptable for the germination, growth and propagation of airborne bacteria. On the other hand, frequent personnel flow and vehicle transportation with product and chickens might also result in increased bacterial concentration in the atmosphere. The highest concentration of airborne bacteria was observed in autumn. In summer, vegetation flourishes and vegetative exudates could kill some bacteria (24) released into the atmosphere under certain conditions, so the bacterial concentration was relative lower than in autumn. In spring, vegetation does not flourish because of the climatic conditions in Beijing, and withered away in winter. There were insufficient growth substrates for bacteria in the atmosphere around the facility, and no significant differences in bacterial concentration were found among the three sampling sites in spring and winter.

Moreover, frequent personnel flow and vehicle transportation with product and chickens might also result in increased bacterial concentration in the atmosphere; therefore, due to vehicles transporting chickens with some bacteria, the highest bacterial concentration was observed in SSC. In GB, a low concentration of airborne bacteria was recorded, which might be attributed to high vegetation coverage, fewer activities such as personnel flow and vehicle transportation and many other environmental factors inhibiting the growth of bacteria. No significant seasonal variation in bacteria concentration was recorded during the year in GB.

Studies showed that some airborne bacteria showed seasonality, corresponding to their seasonal occurrence. *Micrococcus* was predominant during autumn and *Staphylococcus* during summer, while *Bacillus* species were predominant during autumn (17) but no such variation was found in the present study; however, concentration variations of different bacterial groups were found during the year. The variation of *Bacillus* concentration was opposite that of *Micrococcus* and *Staphylococcus*. In different environments, despite the similarity of the bacterial community composition, there was a great discrepancy in the concentration percentages. This demonstrated that the concentration percentages of bacterial groups changed with the environmental and sampling conditions.

In the present study, higher concentrations of *Micrococcus* and *Staphylococcus* were recorded at 9:00 (*P*<0.05), and a lower concentration was found at 13:00 (*P*<0.05). These results were consistent with the previous study. Shaffer reported that the bacterial concentration increased at dawn, then decreased to noon and increased gradually to sunset (25).

In this study, the level of airborne bacteria was investigated comprehensively and systematically among sites at a poultry-slaughtering facility and identified highly contaminated areas at this outdoor facility. In these high-contaminated areas, we should take effective measures to control air pollution, improve environment quality and then protect the health of the personnel. The study has important theoretical and practical significance for air pollution control and improvement of the quality of the environment. Further research is required to identify the level of other biological particles, such as fungi, actinomycetes, viruses and so on at the poultry-slaughtering facility to evaluate airborne microbial communities comprehensively.

## Conclusions

In this study, an investigation of airborne bacteria at an outdoor slaughtering facility was conducted in Beijing. It can be concluded that (i) the prevalent bacterial groups from all the sampling sites were *Micrococcus*, *Staphylococcus*, *Bacillus*, *Corynebacterium* and *Pseudomonas*. The genus of highest concentration was *Micrococcus*; (ii) In different areas, despite the similarity of the bacterial community composition, there was a great discrepancy among the concentration percentages; (iii) There was high bacterial concentration in areas with high vehicle transportation and personnel flow, where the concentration of airborne bacteria might vary according to the seasonal occurrence and was higher in summer and autumn, but lower in spring and winter.

## Figures and Tables

**Table 1:** General situation of sampling sites

Sampling sites	Functional type	Vehicle and personnel	Greenbelt coverage rate	Air pollution
SSC	Shed used to stay chicken waiting for slaughtering	A few transport vehicles with chickens about 10 times h^−1^	~50%	Serious air pollution
EPV	Entrances to personnel and transport vehicles with products	Flow of transport vehicles, and some flow of personnel at commuting time	<5%	A little air pollution
GB	Green belt	Little flow of vehicles and personnel	>95%	Little air pollution

**Table 2:** Culturable airborne bacterial populations collected by FA-1 sampler

Functional areas/genera/species	Functional area		

	SSC (%)	EPV (%)	GB (%)
Gram-positive	82.3	83.7	86.8
*Actinomyces*	1.21	0.41	0.39
*Aerococcus*	0.4	0.41	—
*Arcanobacterium*	—	1.22	1.16
*Arthrobacter*	1.21	0.81	0.39
*Bacillus*	5.65	8.94	6.2
*Brevibacterium*	2.82	5.28	3.1
*Camobacterium*	1.21	0.81	0.39
*Cellulomonas*	0.81	1.22	1.55
*Cellulodimicrobium*	0.4	1.22	0.78
*Clavibacter*	0.81	0.41	1.94
*Corynebacterium*	3.23	4.88	3.88
*Curtobacterium*	2.81	1.63	1.55
*Deinococcus*	1.98	—	0.78
*Dermabacter*	0.4	—	—
*Enterococcus*	0.4	0.41	0.78
*Gardnerella*	—	0.41	—
*Gemella*	0.4	—	1.94
*Gordonia*	0.81	2.03	0.39
*Kocuria*	0.4	1.22	—
*Kurthia*	—	—	0.39
*Kytococcus*	0.81	0.81	—
*Leuconostoc*	0.4	—	—
*Macrococcus*	2.02	1.63	1.55
*Microbacterium*	2.02	1.63	4.26
*Micrococcus*	26.61	35.52	20.54
*Pediococcus*	1.21	0.41	1.94
*Rathaybacter*	0.4	—	—
*Rhodocococcus*	0.4	2.03	1.94
*Staphylococcus*	10.48	8.94	16.28
*Streptococcus*	1.21	1.22	0.39
*Vagococcus*	4.03	0.41	3.1
No identification	7.66	2.85	10.85
Gram-negative	17.7	16.3	13.2
*Achromobacter*	—	—	0.78
*Acinetobacter*	0.81	0.41	—
*Aeromonas*	1.61	—	—
*Brevundimonas*	0.81	—	0.78
*Buttiauxella*	0.4	0.41	—
*Escherichia*	—	0.41	—
*Flavobacterium*	0.4	—	—
*Pantoea*	0.81	1.63	1.94
*Pasteurella*	2.02	0.81	—
*Phyllobacterium*	—	—	0.39
*Pseudomonas*	2.82	4.47	5.04
*Rahnella*	0.4	0.41	0.39
*Ralstonia*	—	—	0.39
*Vibrio*	0.8	2.03	1.16
*Xanthomonas*	—	0.41	—
*Yersinia*	—	—	0.39
No identification	6.85	5.28	1.94

**Table 3:** Concentration data for total airborne bacteria at the three sampling sites

Sampling site	Mean (CFU m^−3^)	Median (CFU m^−3^)	Minimal level (CFU m^−3^)	Maximal level (CFU m^−3^)
SSC	2,714	1,546	142	17,876
EPV	2,664	1,676	188	27,920
GB	1,582	1,172	48	10,658
General	2,330	1,420	48	27,920
